# Self-guided CcARM pogramme-COVID 19-March 2020. Complex case and recovery management framework (the CcARM*) - a quality improvement project

**DOI:** 10.1192/bjo.2021.516

**Published:** 2021-06-18

**Authors:** Syeda Hasan, Mark Spurrell

**Affiliations:** 1 GMMH NHS Foundation Trust; 2 Merseycare NHS Foundation Trust

## Abstract

**Aims:**

During the recent lockdown, it was difficult for those with complex needs associated with learning disability and autism to source timely support. Despite the challenges posed by the COVID-19 epidemic, several resourceful initiatives were implemented, across the clinical landscape

The Self-guided CCaRM Programme was developed as a format for on-line workshops with those concerned. The expectation was to reframe support already there, and streamline further support to best effect.

**Method:**

This programme evolved from the Complex Case and Recovery Management Framework (The CCaRM*), developed within Merseycare Specialist LD Services. This value-based platform was being used by the Specialist Support Team (SST) to support people in the community with LD and Autism with complex needs. With lockdown constraints, the service became reliant on working indirectly through family and carers.

Primary Driver:

- 1:The priority during the lockdown was to make sure how quickly to carry on functioning ,when everyone was distant from each other, and not been able to see people who have Learning Disability & Autism with complex needs.
- 2: Bringing CCARM to the people as a internet based intervention as CcARM was successfully practice with specialist services.
- 3: To provide a format for service users and then career to better review and reframe the care needs, to better effect for themselves

During the recent lockdown, for those with complex needs associated with learning disability and autism:

It was difficult for people to source timely support for themselves.

It was difficult for specialist teams to reach them with useful advice

Secondary Drivers:

- 1:To reframe support already there and to streamline farther support to best effect.
- 2:Increase Engagement:
- 3:Ensure Accessibility
- 4:Continuing workshops through COVID-Pandemic with no gaps in between-in first PDSA cycle

Change Ideas

- 1: The approach to counter the impact of Lockdown in a critical area
- 2: To adapt the CCARM framework to the online environment.
- 3: Simplification to improve over all engagement within the process

A skype-based workshop was convened for all relevant parties. In advance, attendees reviewed concerns using the 6 self-guided CCaRM headings, in line with the original CCaRM, as follows:

- Having a good circle of support
- Having a good shared understanding
- Having clear problem areas thought about
- Social Participation and Living a Good Life
- Keeping people safe and well
- Making progress

For each theme, areas of strengths, concerns, and possible fresh approaches were explored. Subsequently, collaborative care plans were refreshed accordingly.

**Result:**

There were 8 such workshops conducted in first PDSA cycle . Participants included support staff and family members, though no service users in this period. All gave positive feedback: that the experience helped with understanding and confidence in roles, and generated fresh ideas to try.

**Conclusion:**

- This approach helped counter the impact of lockdown in a critical area.
- It was interesting to adapt the CCaRM framework to the online environment.
- The perforce simplification seemed to improve the engagement of carers
- Further work needs to explore potential service user involvement also, and to evaluate the approach longer term.

